# Behavioral and economic traits reflect distinct resource acquisition strategies in tendril vines and stem twining vines

**DOI:** 10.1002/ece3.70271

**Published:** 2024-09-20

**Authors:** Hua Bai, Yingzheng Ji, Xueqing Wang, Zhi Liu, Zhe Zhou, Ming Yue, Yaoxin Guo

**Affiliations:** ^1^ Key Laboratory of Resource Biology and Biotechnology in Western China (Ministry of Education) Northwest University Xi'an China

**Keywords:** climbing mechanism, ecological difference, plant economic spectrum, resource acquisition, vines

## Abstract

Climbing plants are important components of tropical and many temperate forest ecosystems. Current studies regard climbing plants as a single ecological plant type and ignore the ecological differences resulting from their climbing mechanisms, which may lead to a misrepresentation of the role of climbing plants in forest dynamics. Based on behavioral traits and economic traits of climbing plants, we test the hypothesis that tendril climbers and stem twiners are characterized by different resource acquisition strategies. We quantified and compared 4 behavioral traits and 7 economic traits of four stem twining vines and four tendril vines in a temperate oak forest and further tested their differences in resource acquisition strategy. Our study found that tendril vines were scattered in a group distinct from stem twining vines along the first axes of the principal component analysis using four behavioral traits and seven economic traits, being located at the more acquisitive end with more hosts, a larger distance to length ratio of stem, higher leaf and root nitrogen concentrations, and lower leaf carbon content, while stem twining vines showed the opposite trends. These results indicate that tendril vines have a more acquisitive strategy than stem twining vines. The findings suggest a functional variability among the different climbing mechanisms, and which should be accounted for in future studies.

## Graphical abstract

Behavioral traits were corresponded with economic traits in reflecting resource acquisition strategy of climbing plants. Tendril vines showed a more acquisitive strategy than stem twining vines, and the functional differences resulting from climbing mechanisms should be accounted for in future studies on climbing plants under global change.

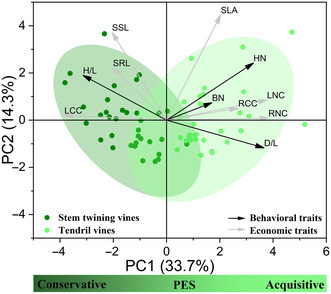

## 1 INTRODUCTION

Climbing plants form an important functional group in numerous forest ecosystems globally and play a vital role in various aspects of forest structure and function (Duran et al., 2015; Poulsen et al., 2017; Schnitzer & Bongers, 2002). Moreover, an increasing number of studies over the past decades have suggested that climbing plants are increasing in abundance and biomass relative to free‐standing plants due to worldwide climate change and forest disturbance (Buckton et al., 2019; Schnitzer & Bongers, 2011; Venegas‐González et al., 2020). To reveal the specific mechanism responsible for disproportionate changes in climbers and free‐standing plants, most studies that investigated the functional traits of climbing plants considered them as a uniform group, lianas (woody climbers), or vines (herbaceous climbers) to contrast them with free‐standing plants (Collins et al., 2016; Medina‐Vega, Bongers, Poorter, et al., 2021; Zhu & Cao, 2010), and a few focused or test the differences among climbers differing in their climbing mechanisms (Medina‐Vega, Bongers, Schnitzer, & Sterck, 2021; Zhou et al., 2022). Consequently, whether different climbing mechanisms are related to specific ecological strategies is still unclear.

Although climbers share a similar growth form, they are far from a homogeneous group of species. Climbing plants have evolved multiple mechanisms for climbing, e.g., twiner (twining by branch or stem), tendrillar (grasping by tendrils), root climber (clinging by adventitious roots), and scrambler (leaning on surrounding structures; Putz, 1984). Some studies have suggested that the climbing mechanism is a trait associated with the distribution of climbers in varying forest environments (Gentry, 1991; Putz & Holbrook, 1991). For example, Dewalt et al. (2000) observed that the relative abundance of stem twiners in a tropical rainforest increased with forest stand age, and that of tendril climbers decreased with forest stand age. Durigon et al. (2014) reported that tendril climbers are more frequent in temperate areas than in tropical and subtropical areas. Recently, Villagra et al. (2021) found that scramblers were more abundant in open canopy plots than that in closed canopy plots, while stem twiners were more abundant in closed canopy plots. These studies suggest that different climbing mechanisms could lead to different trajectories in response to abiotic and biotic environments, and thus develop different ecological strategies. The recognition of ecological differences among climbing mechanisms may be essential for understanding the spatial and temporal abundance patterns of climbing plants. In particular, testing ecological differences among climbing mechanisms within a forest type is also important to understand the coexistence of different climbers given that interspecific ecological differences can drive their niche differentiation, and therefore can decrease the interspecific competition (Bongers et al., 2005; Gallagher & Leishman, 2012).

Among plants with diverse climbing mechanisms, stem twiners and tendril climbers are most common in tropical (Anbarashan & Parthasarathy, 2013; Cai & Bongers, 2007) and extratropical ecosystems (Guo et al., 2012; Leicht‐Young et al., 2010), which piqued the interests of botanists since Darwin's extensive study of climbing plants in the late 1800s (Darwin, 1876). Stem twiners and tendril climbers are characterized by contrasting behaviors for light acquisition. Stem twiners generally produce one main stem and twine one or two supports to scramble vertically gain access to light, whereas tendril climbers develop specialized organs, i.e., tendrils, for climbing, and thus can grasp multiple supports to maximize their light capture (Cai & Song, 2005; Gianoli, 2015). The more active behavior traits of tendril climbers suggest that tendril climbers may be more acquisitive in ecological strategy than stem twining climbers, and thus were observed to increase in high‐light conditions, such as such as early successional stands, canopy gaps, or forest edges (Cai & Song, 2005; Campbell et al., 2018; Malizia & Grau, 2008).

In addition to behavioral traits, plant economic traits, a subset of plant functional traits directly involved in the acquisition, processing and conservation of resources, are suitable tools for describing the various ecological strategies of plants in response to abiotic and biotic determinants (McGill et al., 2006; Reich, 2014). According to the plant economic spectrum (PES), there is always a trade‐off in plant economic traits between plants exhibiting fast resource acquisition on the one hand and conservation of resources on the other (Chave et al., 2009; Reich, 2014; Wright et al., 2004). For example, plants with a resource‐acquisitive strategy usually have acquisitive economic traits, such as high specific leaf area (SLA; leaf area (LA) per unit of dry mass; cm^2^ g^−1^) and leaf nitrogen concentrations (LNC) to support high photosynthetic capacity (Wright et al., 2004) and high specific root length (SRL; root length per unit of dry mass, cm g^−1^) and root nitrogen concentration (RNC) to facilitate the rapid acquisition of belowground resources. In contrast, plants with low SLA, LNC, SRL and RNC tend to be more conservative in their resource acquisition strategy. Therefore, tendril climbers may be characterized by more acquisitive traits than stem twining climbers to keep consistent with active behavioral traits (Dias et al., 2024). Recently, the study of Wang (2020) showed that a tendril liana (*Vitis amurensis*) had higher LNC, SLA, and lower leaf C/N than those of two stem twining lianas (*Actinidia kolomikta* and *Schisandra chinensis*), suggesting that tendril climbers may be more acquisitive in resource acquisition than tendril climbers. However, current knowledge about the ecological differences between the two climbing habits remain very limited except for this one study.

Therefore, in the present study, we conducted a comparative study of four stem twining vines and four tendril vines in a warm temperate forest of China based on behavior and economic traits to examine how the resource acquisition strategies differ between the two common climbing modes within a forest type. Based on the current knowledge on the ecology of climbing plants, we hypothesized that tendril and stem twining vines have contrasting behavior and economic traits, and tendril vines are more acquisitive in resource acquisition than stem twining vines.

## 2 MATERIALS AND METHODS

### 2.1 Study site and species selection

The study site was located in Taibai Mountain (Mt. Taibai) of the Qinling Mountains (Mts. Qinling) (33.825°–34.136°N, 107.689°–107.861°E), Shaanxi Province, China. Mts. Qinling runs in an east–west direction in Central China and forms a transitional zone between northern subtropical and warm temperate zones, which make this location a global biodiversity hotspot. Mt. Taibai is located on the northern slope of the Mts. Qinling and therefore has a warm temperate climate with hot and rainy summers and cold and dry winters. The mean annual precipitation is 751.8 mm and mainly occurs from July to September. The mean annual temperature is 7.6°C, the mean temperature of the coldest month is −5.0°C, and the mean temperature of the hottest month is 19.1°C (Ren et al., 2020).

As the highest mountain of the Mts. Qinling, Mt. Taibai has a large elevation span (780–3767 m) and evident vertical changes in vegetation types. Natural vegetation types along the elevational gradient include oak forests (*Quercus* sp.; <2200 m), birch forests (*Betula* sp.) (2300–2800 m), fir forests (*Abies* sp.) (2800–3200 m), larch forests (*Larix* sp.; 3000–3400 m), and alpine shrubs (>3400 m; Ren et al., 2020). Climbing plants, especially twining and tendril vines, are abundant in the oak forests due to the warm climate and frequent disturbances (Zhou et al., 2020), which provide a natural platform for the comparative study between the two climbing mechanisms.

In the oak forests, we investigated and selected all mature and healthy‐looking (without signs of herbivory or pathogens) individual vines in stem twining and tendril climbing mechanisms along line transects with 100 m altitudinal intervals. In order to guarantee enough individuals to collect samples, we selected four twining vines and four tendril vines which are common in the forest for the estimation of behavioral and economic traits. The eight species belonged to 6 families (Table 1). Finally, we selected 35 individuals from four twining vines and 31 individuals from tendril vines, and each species with at least four mature and well‐grown individuals.

**TABLE 1:** Species name, family, climbing mechanism, and the number of individuals of vines collected in the present study.

Species	Family	Climbing mechanism	Individuals
*Amphicarpaea edgeworthii*	Fabaceae	Stem twining	13
*Dioscorea nipponica*	Dioscoreaceae	Stem twining	14
*Paederia scandens*	Rubiaceae	Stem twining	4
*Aconitum hemsleyanum*	Ranunculaceae	Stem twining	4
*Schizopepon dioicus*	Cucurbitaceae	Tendril	4
*Thladiantha dubia*	Cucurbitaceae	Tendril	7
*Gynostemma pentaphyllum*	Cucurbitaceae	Tendril	9
*Smilax riparia*	Liliaceae	Tendril	11

### 2.2 Behavioral trait measurements

**FIGURE 1:**
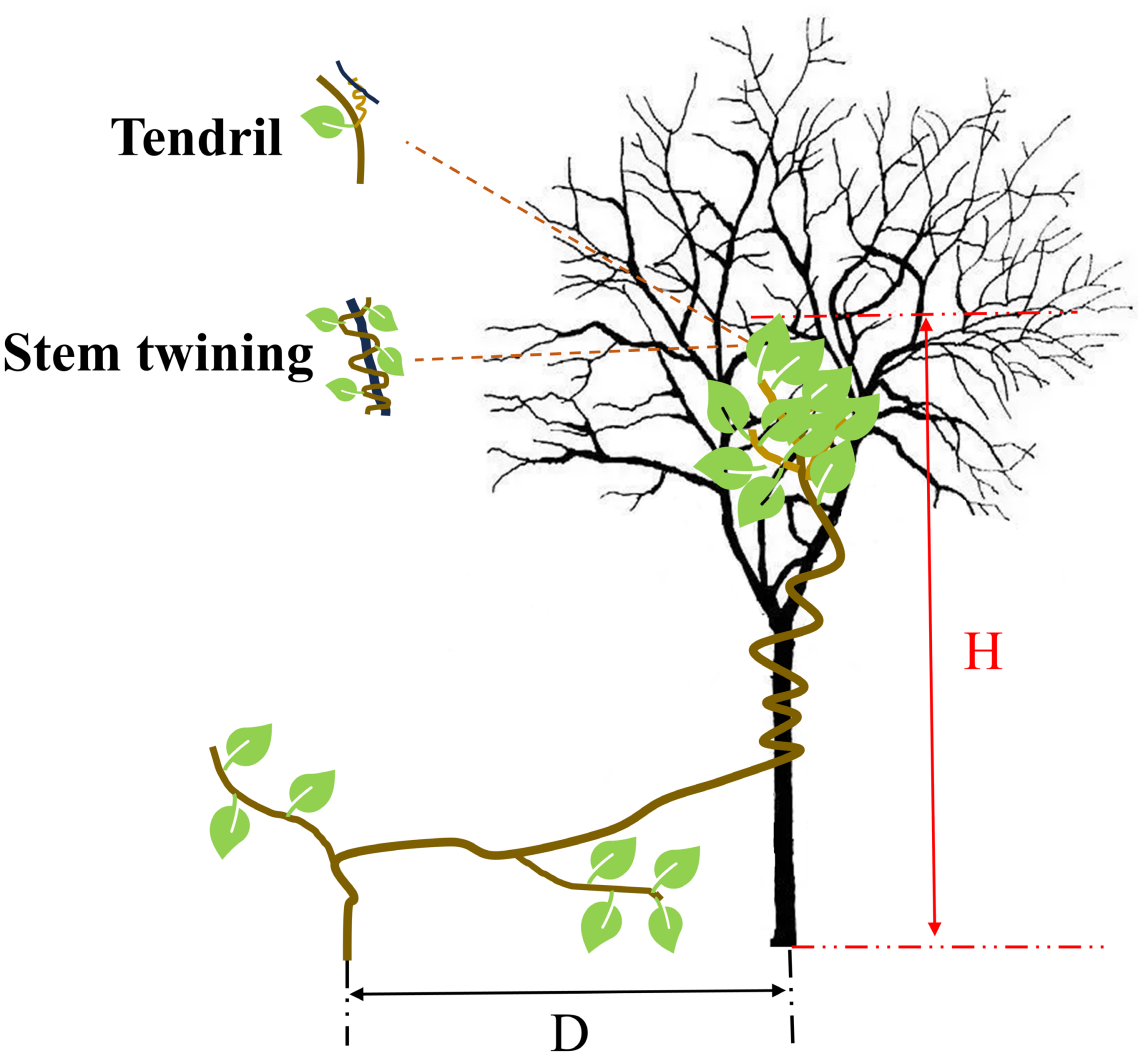
Vertically expanding height (H) and horizontally expanding distance (D) of vines' shoot on a support.

For each individual vine, we measured four behavioral traits concerning space expansion and resource acquisition, including the branch number (BN), host number (HN), height‐to‐length ratio (H/L), and the distance‐to‐length ratio (*D*/*L*; Table 2). The BN was counted as the branching number of main stem for each individual vine. The HN was counted as the number of plants each individual vine attached to. The *H*/*L* ratio was defined as the ratio of climbing height (*H*) to stem length (*L*) and used to assess the vertically expanding ability of vines (Hu & Li, 2014). The *D*/*L* was defined as the ratio of the horizontal expanding distance (from the base to the furthest point that vines can reach) (*D*) to stem length (*L*) and used to assess the laterally expanding ability of vines (Cai & Song, 2005; Figure 1).

**TABLE 2:** List of the measured traits and their abbreviations, units, and ecological relevance.

Traits	Abbreviation	Units	Ecological relevance
Behavioral traits			
Branch number	BN	—	Expansion
Host number	HN	—	Expansion
Height to length ratio of stem	H/L	cm·cm^−1^	Expanding vertically
Distance to length ratio of stem	D/L	cm·cm^−1^	Expanding laterally
Functional traits			
Specific leaf area	SLA	cm^2^·g^−1^	Resource capture
Leaf carbon content	LCC	mg·g^−1^	Resource capture and defense
Leaf nitrogen content	LNC	mg·g^−1^	Resource capture
Root carbon content	RCC	mg·g^−1^	Resource capture and defense
Root nitrogen content	RNC	mg·g^−1^	Resource capture
Specific root length	SRL	cm·g^−1^	Resource capture
Specific stem length	SSL	cm·g^−1^	Resource capture

### 2.3 Economic trait measurements

In our study, we selected seven plant economic traits concerning plant resource acquisition strategy in three dimensions: leaf, stem, and fine root (Table 2). These traits included the specific leaf area (SLA), mass‐based leaf carbon and nitrogen concentrations (LCC and LNC, respectively), specific stem length (SSL), specific root length (SRL), and the mass‐based root carbon and nitrogen concentrations (RCC and RNC, respectively). For each individual vine, we dug the whole plant and then then divided them into the leaf, stem and root fractions for trait and biomass measurements. Trait measurements were conducted for each individual in accordance with the protocol outlined by Cornelissen et al. (2003) and Pérez‐Harguindeguy et al. (2016).

Measurement of leaf traits: for each individual, we collected at least five complete and mature leaves and further measured the LA with a scanner and Motic Image Plus image processing software. All the collected leaves were then dried at 80°C to constant weight to calculate the SLA. Subsequently, the leaf nitrogen content (LNC) and leaf carbon content (LCC) were measured using an elemental analyzer (EA3000, EURO EA, Milan, Italy).

Measurement of root traits: for each individual, the excavated roots were washed with distilled water, and soil particles were removed from the roots with a brush. After that, 5–10 fine root pieces (diameter ≤2 mm) selected were scanned to measure the length for each plant species and then were dried to constant weight. SRL was calculated as the ratio of fine‐root length to dry weight of fine root. Subsequently, we measured the fine‐root nitrogen content (RNC) and fine‐root carbon content (RCC) using an elemental analyzer (EA3000, EURO EA, Milan, Italy).

Measurement of stem traits: for each individual, we first measured the length of each stem. Subsequently, all the collected stems were dried to constant weight. The specific stem length (SSL) was calculated as the ratio of stem length to stem dry weight.

### 2.4 Data analysis

We used generalized linear mixed effects models evaluate the differences in BN and HN between lianas and trees, and used linear mixed effects models to evaluate the other behavioral and economic traits differences between lianas and trees. In the mixed effects models, the climbing mechanism was a fixed effect, and species was a random effect. To control the effect caused by plant size, we put individual total mass as a confounding effect in the model.

To test the associations between traits and the differences in resource acquisition strategy between twining and tendril vines, we conducted principal component analysis (PCA) on the behavioral and economic traits based on the correlation matrix. PCA was performed to determine the nature and number of relevant axes of functional differentiation between the two climbing modes and further visualize their distribution in the two‐dimensional trait space. For the PCA, we standardized each trait by transforming the normalized trait values into z‐scores. To test the differences in ecological strategies between the two climbing modes, we computed the differences in the first and second component scores between them by one‐way analysis of variance (ANOVA).

All analyses were conducted using R v.4.3.2. PCA were conducted using the package vegan. Mixed effect models were conducted using the package lme4.

## 3 RESULTS

**FIGURE 2:**
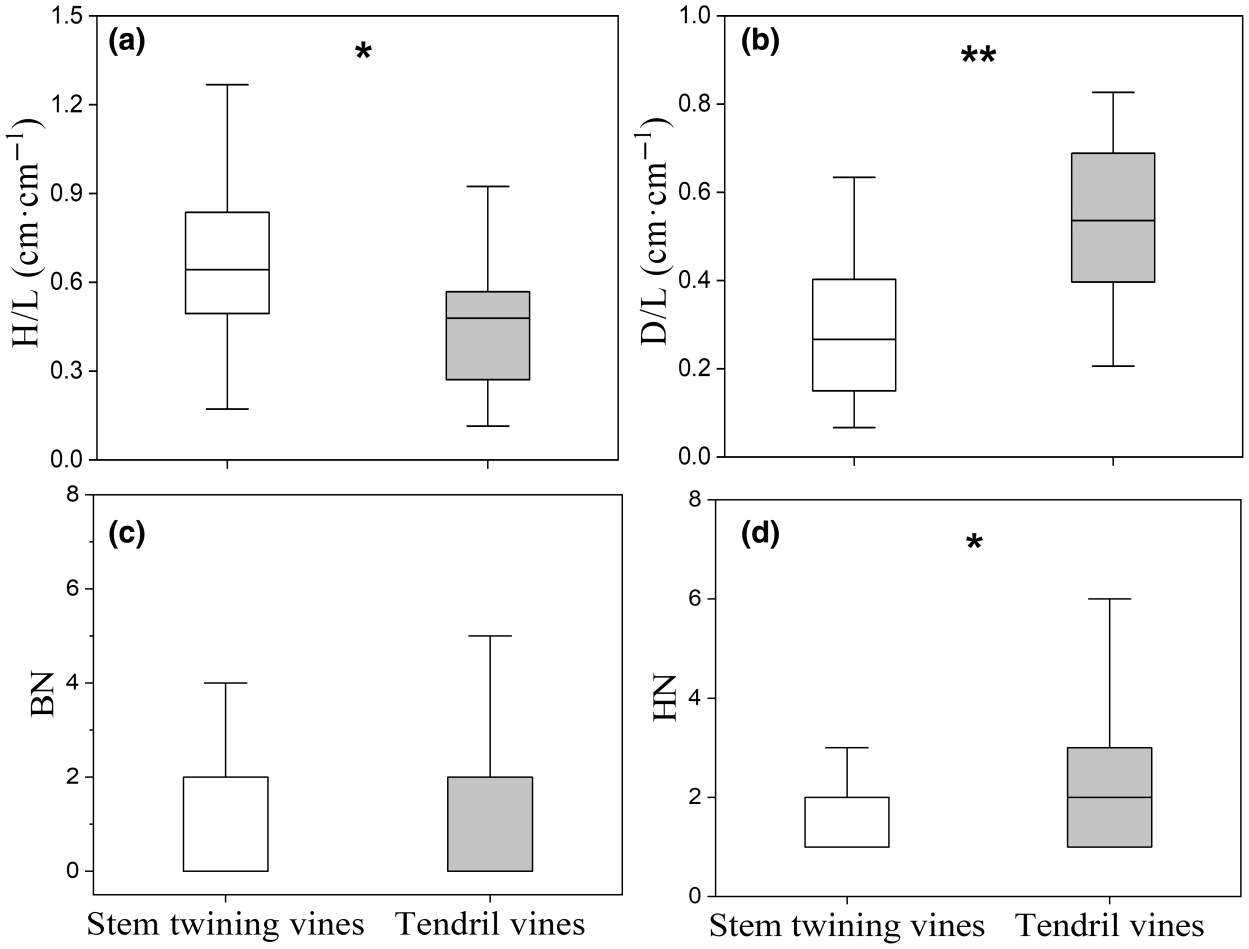
Differences in behavioral traits between stem twining and tendril vines (*Note*: *means *p* < .05, **means *p* < .01).

**FIGURE 3:**
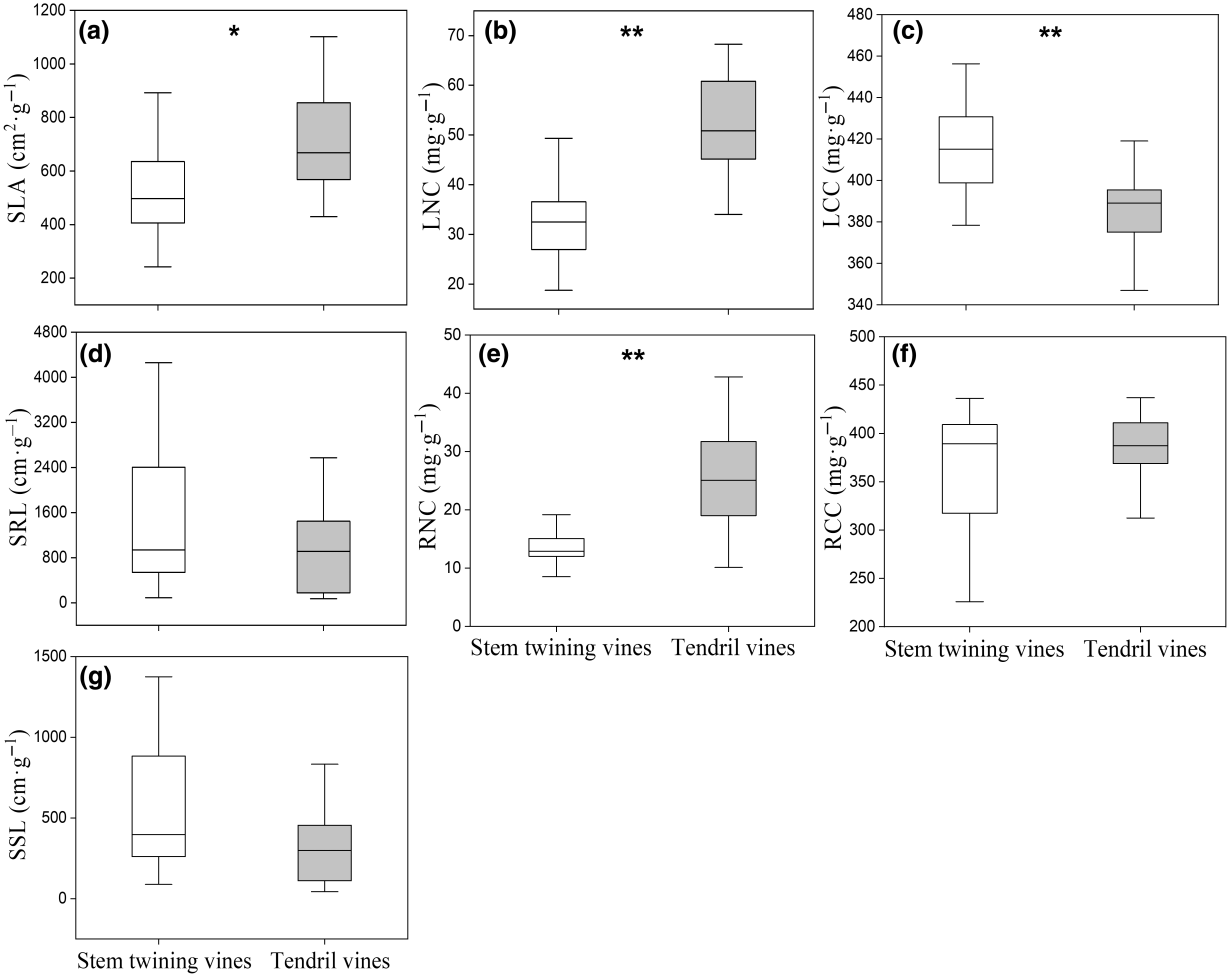
Differences in economic traits between stem twining and tendril vines. (*Note*: *means *p* < .05, **means *p* < .01).

For behavioral traits, tendril climbers showed higher D/L, HN and low H/L than stem twiners, but the latter exhibited a higher H/L and lower D/L and HN (Figure 2a,b,d). BN did not differ between the stem twining and tendril vines but significantly changed with plant size (Table S1). For economic traits, tendril vines had higher SLA, LNC, and RNC and lower LCC than stem twining vines (Figure 3a–c,e). SRL and SSL did not differ between the two climbing mechanisms, but SSL changed significantly with plant size (Table S1).

**FIGURE 4:**
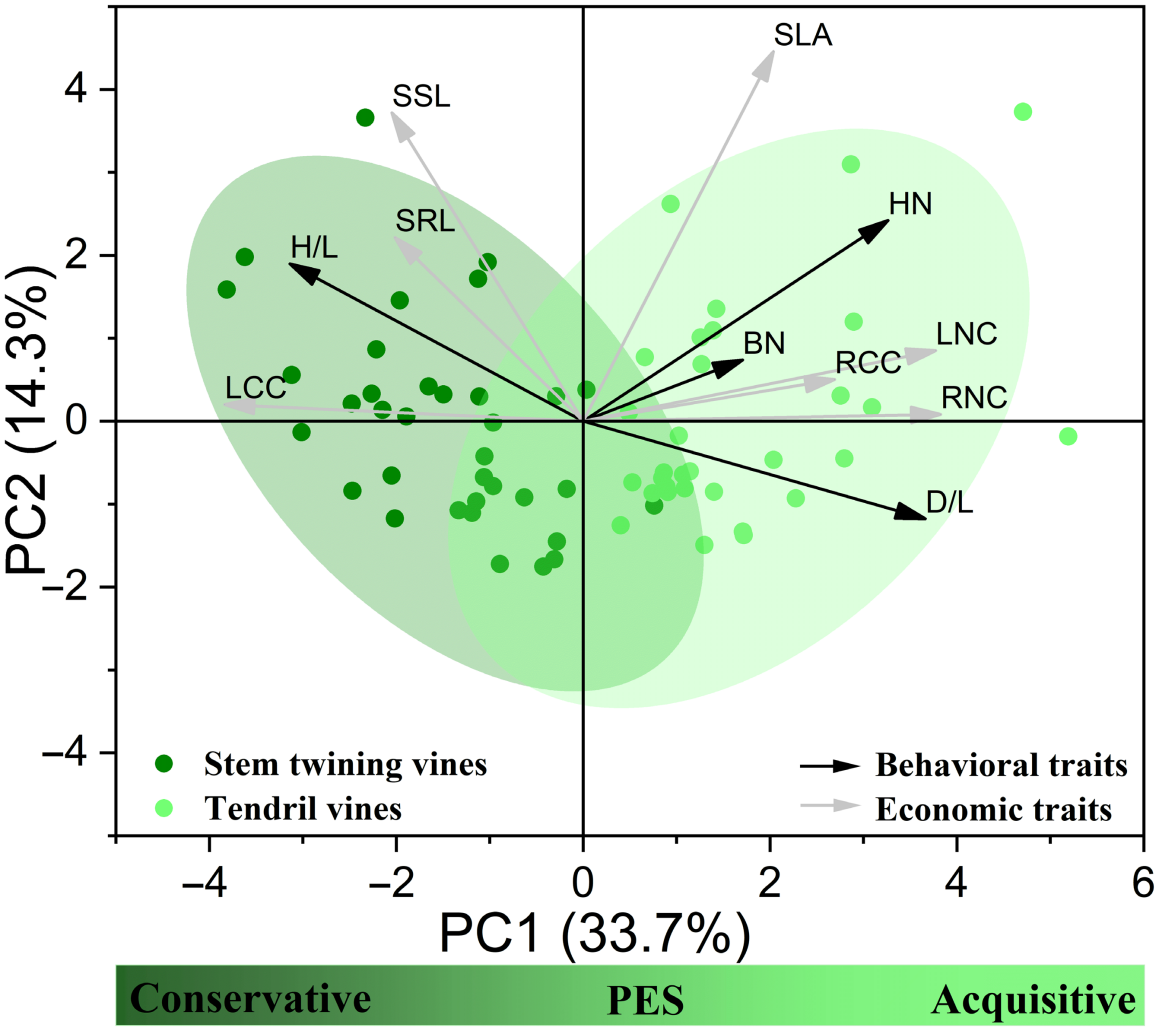
Distribution of stem twining and tendril vines in functional traits space defined by the first two PCA axes.

**FIGURE 5:**
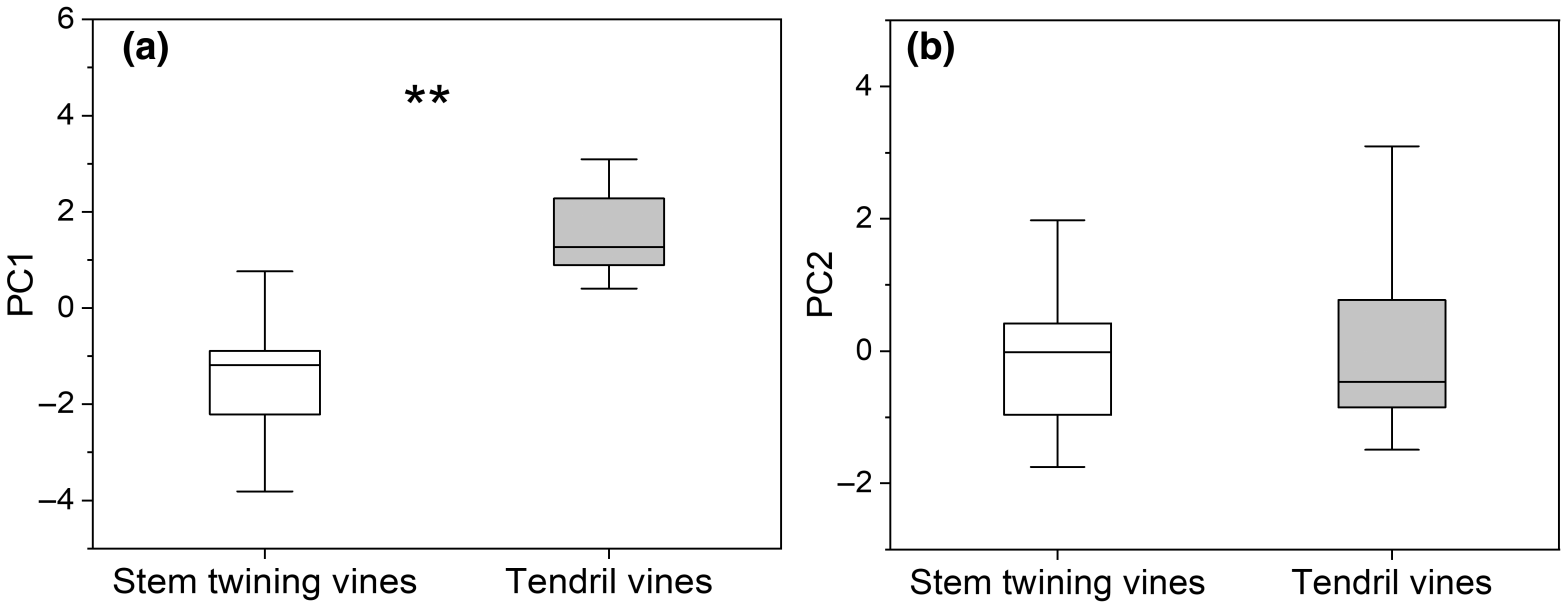
Differences between stem twining and tendril vines in the first and second component scores (*Note*: ** means *p* < .01).

In the PCA that included behavioral traits and economic traits, the first two principal components accounted for 48% of the variance. The first component was associated with *D*/*L*, *H*/*L*, HN, LNC, RNC and LCC, and ran from acquisitive and cheap traits (high *D*/*L*, HN, LNC, RNC and low LCC, *H*/*L*) to conservative traits (high LCC, *H*/*L* and low *D*/*L*, HN, LNC, RNC), while the second component ran from traits characterized by low SSL and SLA to traits characterized by high SSL and SLA (Figure 4, Table S2). Stem twining and tendril vines were significantly separated in the trait space along the first axis of the PCA, with tendril vines occupying the acquisitive side of the gradient and stem twining vines occupying the conservative side (Figures 4 and 5a). However, we did not observe differences between the two climbing mechanisms along the second axis of the PCA (Figure 5b).

## 4 DISCUSSION

We compared the behavior and economic traits of tendril and stem twining vines in a temperate oak forest to test the differences between their ecological strategies. Our data support the hypothesis that tendril vines are more acquisitive in behavior and economic traits than stem twining vines, which would allow them to acquire resources more rapidly and grow faster.

Compared with stem twining vines, tendril vines climbed more hosts, and expand more distant distance per length of stem. In contrast, stem twining vines were adept at climbing vertically depending on one or two hosts and failed to distant expanding. These behavior traits indicate that tendril vines have a greater climbing efficiency and can occupy more extensive horizontal areas than stem twining vines. Congruent with our results, Cai and Song (2005) compared the climbing efficiency of two liana species based on the HN and horizontal extensive area, and also found that individuals of tendril liana (*Ampelopsis cantoniensis*) had greater climbing efficiency than individuals of stem twining liana (*Dalbergia millettii*). The contrasting behavior traits of tendril and stem twining climbers reflected their different behavior strategies in resource acquisition. The higher climbing efficiency and expanding ability in space of tendril climbers than stem twining climbers can make tendril climbers be better at resource acquisition, especially for light capture, in heterogeneous environment (Gianoli, 2015). Therefore, tendril climbers were more acquisitive than stem twining climbers in climbing behavior.

Consistent with behavioral traits, tendril climbers also showed more acquisitive economic traits than stem twiners. In our study, tendril vines had higher SLA, LNC, and RNC and lower LCC than stem twiners. High SLA and LNC and low LCC suggest a great surface area available for photosynthesis and a high photosynthetic rate at the same expenditure of structural C (Wright et al., 2010), and thus, leaves with such properties may be rapid turnover. Similarly, a high RNC can be representative of protein concentration associated with nutrient uptake and assimilation and a high turnover rate of roots (Collins et al., 2016; Sterner & Elser, 2002). Rapid turnover in leaf and root may allow tendril vines to explore above‐ and belowground resource more efficiently and may be less costly to the plant via low C construction costs (Caplan et al., 2014). Comparatively, Wang (2020) found that tendril lianas (*Vitis amurensis*) had higher LNC, SLA and lower leaf C/N than those of two stem twining lianas (*Actinidia kolomikta* and *Schisandra chinensis*), suggesting the functional difference in the two climbing modes. More acquisitive leaf and root traits relative to stem twining vines indicate that tendril climbers employ a rapid resource acquisition strategy than stem twining climbers (Díaz et al., 2016).

An exception was the absence of differences in the SRL between the two groups. According to PES theory, roots with a high nutrient absorption capacity are thin (Luke et al., 2012); thus, a high SRL is commonly associated with a resource‐acquisitive strategy. However, previous studies suggested that different root sizes influence the value of SRL and the coordination between root and leaf traits. Collins et al. (2016) observed that lianas have higher SRL than trees with 0–1 mm roots but with no differences for the 1–2 mm root size class. Based on the data of 55 herbs, Cheng et al. (2016) discovered that the relationship between SRL and SLA changed from a significant positive correlation to a significant negative correlation with the increase in root order rank. In our study, the root order was not strictly determined at ≤2 mm, which may explain the inconsistent SRL with leaf and other root traits reflecting resource acquisition between stem twiners and tendril climbers.

Overall, the differences in behavior and economic traits between tendril and stem twining vines reveal their functional difference resulting from their climbing mechanisms. Acquisitive behavior traits give tendril vines a high foraging capacity in heterogenetic environments (Gianoli, 2015), and meanwhile acquisitive economic traits allow them to acquire resources more rapidly and grow faster (Bai et al., 2020). Therefore, tendril vines may have a high advantage over stem twining vines in the competition for resource in high‐resource conditions. In contrast, stem twiners need to cling on the host and are usually shaded by themselves and the crown of their supports and thus develop more conservative traits to adapt to shade environments (Putz et al., 1989). This can explain why tendril climbers flourish more in early successional or disturbed forests, while stem twiners are more abundant in old‐growth or closed forests (DeWalt et al., 2000; Yuan et al., 2009). In addition, the diverge in behavior and economic traits between tendril and stem twining vines reflect their contrasting adaptive strategies to heterogenetic forest environments, which may improve their spatial separation and reduce the intergroup competition in resource acquisition, thereby may promoting their stable coexistence.

## 5 PROSPECTS BASED ON THIS STUDY

Climbing plants have long been attracting the interest of botanists and ecologists because of their distinctive growth strategies and ecological roles (Darwin, 1876; Isnard & Silk, 2009; Schnitzer & Bongers, 2002). Although numerous trait‐based studies have been carried out on the ecology of climbers, the results on their ecological strategies vary across different studies (Medina‐Vega, Bongers, Poorter, et al., 2021; Zhou et al., 2022). One possible explanation for these different results is that these studies considered climbing plants as a uniform group and ignored the functional differences among species resulting from their climbing mechanisms. Notably, the proportions of different climbing mechanisms vary among various forest environments (DeWalt et al., 2000; Gallagher & Leishman, 2012). Therefore, if differences between groups of climbing plants exist and are disregarded, the role of climbing plants may be misrepresented.

Our study found that tendril and stem twining vines diverged in ecological strategy and provided strong evidence for the functional differences among climbing plants resulting from their climbing mechanisms. It suggests that the type of climbing mechanism is a central characteristic in understanding the functional ecology and distribution of climbing plants and should be accounted for in future studies. In addition, our findings suggest that tendril vines have a more acquisitive strategy than stem twining vines, and therefore whose abundance may strongly depend on forest structure and abiotic environments. With the increase in disturbances in forests worldwide, atmospheric CO_2_ concentration, and N deposition, tendril climbers are more likely to increase their abundance and their ecological effects than stem twining climbers and drive the increase in abundance and biomass of climbing plants. Future studies should attempt to test these hypotheses. More work must be conducted to determine whether our findings in vines can be extended to lianas or beyond the species used in this study across diverse biogeographic regions.

## 6 CONCLUSION

Our study results support the hypothesis that the behavior and economic traits of tendril vines are more indicative of a rapid resource acquisition strategy than those of stem twining vines. The higher number of acquisitive traits of tendril vines may provide them with a competitive advantage under global change scenarios, including forest disturbances, CO_2_ fertilization, and N deposition. Our results provide direct evidence of the ecological differences among species resulting from their climbing mechanisms, which should be accounted for in future studies.

## AUTHOR CONTRIBUTIONS

**Hua Bai:** Data curation (equal); formal analysis (equal); investigation (equal); writing – original draft (lead). **Yingzheng Ji:** Data curation (equal); investigation (equal). **Zhi Liu:** Data curation (equal); investigation (equal). **Xueqing Wang:** Data curation (equal); investigation (equal). **Zhe Zhou:** Data curation (equal); investigation (equal). **Ming Yue:** Conceptualization (equal); writing – review and editing (equal). **Yaoxin Guo:** Conceptualization (lead); funding acquisition (equal); methodology (equal); writing – review and editing (lead).

## CONFLICT OF INTEREST STATEMENT

The authors declare that they have no known competing financial interests or personal relationships that could have appeared to influence the work reported in this paper.

## Supporting information


Table S1.
Table S2.

## Data Availability

Behavioral and economic traits: Dryad doi: https://datadryad.org/stash/share/Fq‐QsdmDBSghlY6Z61Mbe9SMhA9ZjObCa81czpkmhc4.
