# Supplementary material for: Behavioral and economic traits reflect distinct resource acquisition strategies in tendril vines and stem twining vines
Source: Ecol Evol. 2024 Sep 20;14(9):e70271. doi: 10.1002/ece3.70271 (PMC11413626; doi:10.1002/ece3.70271)
Supplement: Supplementary file 1 — Table S1. Table S2. [file ECE3-14-e70271-s001.docx]

**Supplementary Materials:** **Behavioral and economic traits reflect distinct resource acquisition strategies in tendril vines and stem twining vines**

**Table S1** Results of mixed effects models comparing behavioral and economic traits between stem twining and tendril vines.

| Traits | *R*^2^ | Fixed effect | | | |
| --- | --- | --- | --- | --- | --- |
|  |  | Individual size | | Climbing mechanism | |
|  |  | F | *P* | F | *P* |
| H/L | 0.268 | 0.041 | 0.840 | 7.666 | **0.027** |
| D/L | 0.465 | 1.635 | 0.209 | 14.900 | **0.009** |
| BN | 0.576 | 13.286 | **<0.001** | 1.875 | 0.361 |
| HN | 0.353 | 0.652 | 0.437 | 4.661 | **0.041** |
| SLA | 0.419 | 1.153 | 0.287 | 6.889 | **0.041** |
| LNC | 0.708 | 0.183 | 0.673 | 24.409 | **0.005** |
| LCC | 0.628 | 0.035 | 0.851 | 7.021 | **0.034** |
| SRL | 0.392 | 0.116 | 0.734 | 1.121 | 0.334 |
| RNC | 0.493 | 0.078 | 0.779 | 39.942 | **<0.001** |
| RCC | 0.091 | 0.129 | 0.722 | 1.702 | 0.239 |
| SSL | 0.498 | 6.115 | **0.042** | 0.076 | 0.761 |

**Note:** Numbers in bold represent significant differences at *α* = 0.05; For explanation of abbreviations, see Table 2.

**Table S2** Principal component analysis results of behavioral and economic traits for stem twining and tendril vines.

| Traits | PC1 | PC2 | PC3 |
| --- | --- | --- | --- |
| HL | -0.314 | 0.266 | -0.087 |
| DL | 0.366 | -0.165 | 0.071 |
| BN | 0.171 | 0.103 | -0.656 |
| HN | 0.326 | 0.339 | -0.395 |
| SLA | 0.204 | 0.624 | 0.030 |
| LNC | 0.377 | 0.119 | 0.269 |
| LCC | -0.384 | 0.028 | 0.119 |
| SRL | -0.201 | 0.310 | -0.129 |
| RNC | 0.382 | 0.011 | 0.295 |
| RCC | 0.269 | 0.071 | 0.246 |
| SSL | -0.205 | 0.521 | 0.387 |
| %Total | 33.67 | 14.33 | 11.29 |

For explanation of abbreviations, see Table 2.
